# Adaptive Robust Unscented Kalman Filter for AUV Acoustic Navigation

**DOI:** 10.3390/s20010060

**Published:** 2019-12-20

**Authors:** Junting Wang, Tianhe Xu, Zhenjie Wang

**Affiliations:** 1 Institute of Space Science, Shandong University, Weihai 264209, China; 2 School of Geosciences, China University of Petroleum (East China), Qingdao 266580, China

**Keywords:** AUV acoustic navigation, unscented Kalman filter, adaptive filter, Sage-Husa filter, robust estimation

## Abstract

Autonomous underwater vehicle (AUV) acoustic navigation is challenged by unknown system noise and gross errors in the acoustic observations caused by the complex marine environment. Since the classical unscented Kalman filter (UKF) algorithm cannot control the dynamic model biases and resist the influence of gross errors, an adaptive robust UKF based on the Sage-Husa filter and the robust estimation technique is proposed for AUV acoustic navigation. The proposed algorithm compensates the system noise by adopting the Sage-Husa noise estimation technique in an online manner under the condition that the system noise matrices are kept as positive or semi positive. In order to control the influence of gross errors in the acoustic observations, the equivalent gain matrix is constructed to improve the robustness of the adaptive UKF for AUV acoustic navigation based on Huber’s equivalent weight function. The effectiveness of the algorithm is verified by the simulated long baseline positioning experiment of the AUV, as well as the real marine experimental data of the ultrashort baseline positioning of an underwater towed body. The results demonstrate that the adaptive UKF can estimate the system noise through the time-varying noise estimator and avoid the problem of negative definite of the system noise variance matrix. The proposed adaptive robust UKF based on the Sage-Husa filter can further reduce the influence of gross errors while adjusting the system noise, and significantly improve the accuracy and stability of AUV acoustic navigation.

## 1. Introduction

With the development of underwater acoustic communication, energy, control, and navigation technologies, autonomous underwater vehicles (AUVs) have been widely used in submarine detection, ocean environmental monitoring, deep-sea facility laying and monitoring, marine mining, and other applications [1,2]. The autonomous navigation systems for AUVs include acoustic navigation, gravity-magnetic matching navigation, and integrated navigation systems (INS) [3,4]. Compared with gravity-magnetic matching navigation and INS, acoustic navigation does not need to store accurate underwater gravity-magnetic maps in advance, and has a smaller size and lighter weight. Although acoustic navigation technology is challenging due to high costs, difficulty in construction of seabed beacon, and complexity of calibration for obtaining optimal positioning accuracy, it is widely used in AUV navigation as an independent navigation system or as a key part of integrated navigation [5].

For AUV real-time navigation, different filter methods have been developed, including the extended Kalman filter (EKF), unscented Kalman filter (UKF), and particle filter (PF) [6,7]. The EKF uses the first-order Taylor expansion to calculate the Jacobian matrix, which not only increases the complexity of the mathematical computation, but also introduces linearization errors. The PF has good performance under nonlinear conditions, but requires a large number of particles to achieve an optimal filtering effect. At the same time, the degradation of the PF will seriously affect the performance of the algorithm. The UKF utilizes unscented transform (UT) to estimate the system state vector and its covariance matrix, which reduces the impact of linearization errors [8]. Considering both the efficiency and accuracy of these algorithms, the UKF is more suitable for AUV acoustic navigation.

To address the problem of unknown noise statistics in the dynamic and measurement models, different adaptive Kalman filters have been studied, such as maximal post-filter statistics based on Sage-Husa, an adaptive filter based on variance component estimation [9], multiple-model-based adaptive estimation (MMAE) [10], innovation-based adaptive estimation (IAE) [11], and residual-based adaptive estimation (RAE) [12]. In addition, an adaptive Kalman filter based on the attenuation memory method and the limited memory method was proposed to solve the divergence problem of the classical Kalman filter [13,14]. For the Sage-Husa method, to achieve unbiased estimation, measurement and system noise cannot be estimated simultaneously, and the positive or semipositive definite values of the noise matrices cannot be guaranteed, which can cause filter divergence [15,16]. For the IAE and RAE methods, the adaptation is applied directly to the covariance matrices of the measurement and system noises in accordance with the difference of the observation residual or innovation sequence. To realize these methods, the innovation or residual vectors of a certain history window must be known, causing an increment in the storage burden, and the width of the moving window must also be known [17,18]. In the attenuation memory and the limited memory method, it only relies on the adaptive attenuation factor for adjustment and can easily make the filter diverge [14]. Additionally, if the observations contain gross errors, the filter cannot work effectively. The gross error control strategies include outlier detection and robust estimation [19]. In terms of robust estimation, the equivalent weight scheme based on the M-estimation [20] has been widely used. The commonly used robust estimation methods include the Huber method [21], minimal one-norm estimation ($L^{1}$ estimation), minimal p-norm estimation (*L*^P^ estimation) [22], the Danish method, the Hampel method [23], the institute of geodesy and geophysics (IGG) method [24], and other. In recent years, many studies have focused on the robust Kalman filter based on the M-estimation. The robust Kalman filter is widely used in global navigation satellite system (GNSS) positioning and navigation. Yang proposed an adaptive robust Kalman filter that combines the adaptive factor and the institute of geodesy and geophysics III (IGGIII) equivalent weight [25]. Chang proposed an adaptive method with fading memory and a robust method with enhancing memory in the Kalman filter [13]. Yang proposed an adaptive robust Kalman filter based on a global positioning system (GPS)-dead reckoning (DR) integrated navigation system to give more actual and reliable parameter estimation of the maneuvering vehicles [26].

In normal conditions with known initial system noise of the dynamic model and white noise of the measurement model, the conventional UKF can provide good estimation results for AUV navigation [27]. However, if the initial system noise deviates too much from the real noise, or if gross error exists in the observation, the UKF can be greatly influenced, causing inaccurate results or even filter divergence. The conventional UKF cannot adaptively adjust the system noise and control the influence of gross errors. In order to solve the aforementioned problems, this paper proposes an adaptive robust UKF based on the Sage-Husa filter and the robust estimation to adaptively estimate the system noise of the dynamics model and resist the effect of gross errors in the acoustic observations. The paper is organized as follows. We begin by presenting the theory of the UKF in Section 2. Section 3 introduces the theory of the adaptive UKF, as well as the theoretical derivation and algorithm implementation of the adaptive robust UKF. The adaptive UKF and the adaptive robust UKF are verified and analyzed by experimental data in Section 4. Finally, we summarize the significant conclusions in Section 5.

## 2. Unscented Kalman Filter (UKF)

Owing to the nonlinear characteristic of the dynamic and measurement model in AUV navigation and positioning, a nonlinear Kalman filter, such as the UKF, should be adopted instead of the linear Kalman filter. The AUV state equation of the nonlinear system and the acoustic measurement equation can be expressed as:

(1) $$x_{k}=\varphi \left(x_{k-1}\right)+\Gamma \left(\omega_{k-1}\right)$$

(2) $$z_{k}=H\left(x_{k}\right)+\Delta_{k}$$

where $x_{k}$, $x_{k-1}$ are the state vectors of the AUV at times k,k−1 , respectively; $\varphi \left(x_{k-1}\right)$ is the nonlinear state transition matrix; $\omega_{k-1}$ is the system noise; $\Gamma (\omega_{k-1})$ is the system noise distribution matrix; $z_{k}$ is the acoustic observation vector at the time of k; $H\left(x_{k}\right)$ is the nonlinear observation matrix; and $\Delta_{k}$ is the measurement noise. The system noise and the measurement noise with covariance matrices $Q_{k}$, $R_{k}$ are usually assumed to be uncorrelated Gaussian white noise:

(3) $$\{\begin{array}{c} Cov\left[\omega_{k},\omega_{j}\right]=E\left[\omega_{k}\omega_{j}^{T}\right]=Q_{k}\delta_{kj},\;E\left[\omega_{k}\right]=0\;\;\\ Cov\left[\Delta_{k},\Delta_{j}\right]=E\left[\Delta_{k}\Delta_{j}^{T}\right]=R_{k}\delta_{kj},\;E\left[\Delta_{k}\right]=0\;\\ Cov\left[\omega_{k},\Delta_{j}\right]=E\left[\omega_{k}\Delta_{j}^{T}\right]=0 \end{array}$$

where $\delta_{kj}$ is Kronecker delta function.

The basic principle of the UKF is to take sigma points according to the UT in the original state distribution. Therefore, the mean and covariance of these points are equal to those of the original state distribution. Then, these points are substituted into the nonlinear function to get the transformed mean and covariance [28,29]. The standard UKF implementation for state estimation is given as follows.

Step 1: We initialize the state parameter and the corresponding covariance.

(4) $${\hat{x}}_{0}=E\left(x_{0}\right)$$

(5) $$P_{0}=E\left\{\left[x_{0}-{\hat{x}}_{0}\right]{\left[x_{0}-{\hat{x}}_{0}\right]}^{T}\right\}$$

where $x_{0}$, ${\hat{x}}_{0}$ denote the initial and mean state information, including the x coordinate, y coordinate, speed, turning radius, and turning angle of AUV, while $P_{0}$ stands for the initial covariance matrix.

Step 2: The parameters are then updated.

The sigma points and weights are calculated as follows:

(6) $$\chi_{k}=\left\{{\hat{x}}_{k},{\hat{x}}_{k}+{\left[\sqrt{\left(n+\lambda \right)P_{x,k}}\right]}_{i},{\hat{x}}_{k}-{\left[\sqrt{\left(n+\lambda \right)P_{x,k}}\right]}_{i}\right\},\;\left(i=1,\cdots ,n\right)$$

(7) $$W_{i}^{\left(m\right)}=W_{i}^{\left(c\right)}=\{\begin{array}{c} \lambda /\left(n+\lambda \right),\;\;i=0\\ 1/\left[2\left(n+\lambda \right)\right],\;\;i=1,\cdots ,2n \end{array}$$

where $W_{i}^{\left(m\right)}$, $W_{i}^{\left(c\right)}$ denote the weighted values of the mean and covariance, respectively; $x\sim N\left(\bar{x},P_{x}\right)$, $\;\bar{x}$, $P_{x}$ denote the mean and covariance of coordinates, respectively; λ is the proportional parameter; and $\chi_{i,k}$ is the weighted point that approximates the random variable distribution.

We calculate the prediction sigma points, the state value, and the covariance matrix as

(8) $$\chi_{i,k}^{-}=\varphi \left[\chi_{i,k}\right]\;\left(i=1,\cdots ,2n\right)$$

(9) $${\hat{x}}_{k}^{-}=\overset{2n}{\underset{i=0}{\sum }}W_{i}^{\left(m\right)}\chi_{i,k}^{-}$$

(10) $$P_{x,k}^{-}=\overset{2n}{\underset{i=0}{\sum }}W_{i}^{\left(c\right)}[\chi_{i,k}^{-}-{\hat{x}}_{k}^{-}]{\left[\chi_{i,k}^{-}-{\hat{x}}_{k}^{-}\right]}^{T}+Q_{k}$$

where $Q_{k}$ denotes the state noise covariance matrix.

Step 3: The prediction value of measurement is computed.

(11) $${\hat{z}}_{k}^{-}=\overset{2n}{\underset{i=0}{\sum }}W_{i}^{\left(m\right)}H[\chi_{i,k}^{-}]\;\;$$

Step 4: We calculate the variance and covariance matrix.

(12) $$P_{z,k}=\overset{2n}{\underset{i=0}{\sum }}W_{i}^{\left(c\right)}\{H[\chi_{i,k}^{-}-{\hat{z}}_{k}^{-}]\}{\left\{H\left[\chi_{i,k}^{-}-{\hat{z}}_{k}^{-}\right]\right\}}^{T}+R_{k}$$

(13) $$P_{xz,k}=\overset{2n}{\underset{i=0}{\sum }}W_{i}^{\left(c\right)}\{\chi_{i,k}^{-}-{\hat{x}}_{k}^{-}\}{\left\{H\left[\chi_{i,k}^{-}-{\hat{z}}_{k}^{-}\right]\right\}}^{T}$$

where $R_{k}$ denotes the measurement noise covariance matrix

Step 5: The gain of the Kalman filter is calculated.

(14) $$K_{k}=P_{xz,k}P_{z,k}^{-1}$$

Step 6: We update the state vector and the covariance matrix in the following.

(15) $${\hat{x}}_{k}={\hat{x}}_{k}^{-}+K_{k}\left[{\hat{z}}_{k}-{\hat{z}}_{k}^{-}\right]$$

(16) $$P_{x}=P_{x,k}^{-}-K_{k}P_{z,k}K_{k}^{T}$$

## 3. Adaptive Robust Kalman Filter

### 3.1. Adaptive UKF Based on the Sage-Husa Filter

When the system noise is unknown, the classical UKF cannot adjust the system noise. We focus on the above problem to study the adaptive UKF algorithm based on the Sage-Husa filter. The Sage-Husa filter adaptively estimates the system and measurement noise through the time-varying noise estimator, including the mean of the system noise vector ${\hat{q}}_{k}$, the system noise variance matrix ${\hat{Q}}_{k}$, the mean of the measurement noise vector ${\hat{r}}_{k}$, and the measurement noise variance matrix ${\hat{R}}_{k}$ [30,31]. The estimators of the Sage-Husa filter can be expressed as [32]:

(17) $${\hat{q}}_{k}=\left(1-d_{k-1}\right){\hat{q}}_{k-1}+d_{k-1}\left({\hat{x}}_{k}-{\hat{x}}_{k}^{-}\right)$$

(18) $${\hat{Q}}_{k}=\left(1-d_{k-1}\right){\hat{Q}}_{k-1}+d_{k-1}\left(K_{k}v_{k}v_{k}^{T}K_{k}^{T}+P_{k}-\overset{2n}{\underset{i=0}{\sum }}W_{i}^{\left(c\right)}\left[\chi_{i,k}^{-}-{\hat{x}}_{k}^{-}\right]{\left[\chi_{i,k}^{-}-{\hat{x}}_{k}^{-}\right]}^{T}\right)$$

(19) $${\hat{r}}_{k}=\left(1-d_{k-1}\right){\hat{r}}_{k-1}+d_{k-1}\left(z_{k}-{\hat{z}}_{k}^{-}\right)$$

(20) $${\hat{R}}_{k}=\left(1-d_{k-1}\right){\hat{R}}_{k-1}+d_{k-1}\left(v_{k}v_{k}^{T}-\overset{2n}{\underset{i=0}{\sum }}W_{i}^{\left(c\right)}\left\{H\left[\chi_{i,k}^{-}-{\hat{z}}_{k}^{-}\right]\right\}{\left\{H\left[\chi_{i,k}^{-}-{\hat{z}}_{k}^{-}\right]\right\}}^{T}\right)$$

where $v_{k}=z_{k}-{\hat{z}}_{k}^{-}-{\hat{r}}_{k-1}$ is the one-step predicting residual, $d_{k-1}=\left(1-b\right)/\left(1-b^{k}\right),\left(j=0,1\cdots ,k-1\right)$, and b is the forgetting factor with the range of 0.95<b<0.99 [33].

According to Equations (18) and (20), the Sage-Husa filter based on the time-varying noise estimation can theoretically obtain both the system and measurement noise matrices. However, the deviation of residual vector $v_{k}$ affects the calculation of ${\hat{Q}}_{k}$,$\;{\hat{R}}_{k}$ simultaneously, easily leading to filter divergence. Hence, Equations (18) and (20) cannot be used simultaneously. Therefore, the adaptive UKF algorithm studied in this paper only adjusts the system noise using Equations (17) and (18). The covariance matrix in Equation (10) is rewritten as

(21) $$P_{x,k}^{-}=\overset{2n}{\underset{i=0}{\sum }}W_{i}^{\left(c\right)}\left[\chi_{i,k}^{-}-{\hat{x}}_{k}^{-}\right]{\left[\chi_{i,k}^{-}-{\hat{x}}_{k}^{-}\right]}^{T}+{\hat{Q}}_{k}$$

In addition, Equation (18) cannot guarantee the system noise variance matrix ${\hat{Q}}_{k}$ or the semi positive or positive definite values, which may give it a negative definite value. The eigenvalue eig of the system noise variance matrix ${\hat{Q}}_{k}$ is calculated. If the eig≥0, the ${\hat{Q}}_{k}$ is calculated by Equation (18), otherwise ${\hat{Q}}_{k}$ is calculated by Equation (22).

(22) $${\hat{Q}}_{k}=\left(1-d_{k-1}\right){\hat{Q}}_{k-1}+d_{k-1}\left(K_{k}v_{k}v_{k}^{T}K_{k}^{T}+P_{k}\right)$$

### 3.2. Adaptive Robust UKF Algorithm 

The standard Kalman filter requires the system and measurement noise to be zero-mean white noise. Owing to the complexity of the marine environment, gross errors inevitably exist in the underwater acoustic observation [34]. Therefore, robust estimation is usually used to reduce the influence of gross errors on positioning results.

In underwater acoustic positioning, when the observation contains gross error, the measurement equation should be

(23) $${\tilde{z}}_{k}=H\left(x_{k}\right)+G_{k}\varepsilon_{k}+\Delta_{k}$$

where $G_{k}$ is the interference matrix of the gross error, and $\varepsilon_{k}$ denotes the gross error.

If the standard UKF is still used for calculation, the prediction residual with gross error is as follows:

(24) $${\tilde{v}}_{k}={\tilde{z}}_{k}-H\left(x_{k}\right)=v_{k}+G_{k}\varepsilon_{k}$$

(25) $$v_{k}=z_{k}-{\hat{z}}_{k}^{-}$$

(26) $${\tilde{v}}_{k}={\tilde{z}}_{k}-{\hat{z}}_{k}^{-}$$

where $v_{k}$, ${\tilde{v}}_{k}$ are the prediction residual vectors without gross error and with gross error, respectively.

The gross error is fully reflected in the prediction residual, so the state vector is

(27) $${\hat{x}}_{k}={\hat{x}}_{k}^{-}+K_{k}{\tilde{v}}_{k}$$

The gross error in the observation affects the state vector ${\hat{x}}_{k}$ through the gain matrix $K_{k}$. Therefore, the influence of the gross error on the state estimate is significantly reduced or even eliminated by using the prediction residual to constrain the gain matrix. Based on Huber’s constructed equivalent weight function [21], the equivalent gain matrix is constructed as:

(28) $${\bar{K}}_{K}=\{\begin{array}{c} \;\;\;K_{k\;\;}\;\;\left|S_{k}\right|<c\\ K_{k}/\left|S_{k}\right|\;\;\left|S_{k}\right|\geq c \end{array}$$

(29) $$S_{k}=v_{k}/\sigma_{0}$$

(30) $$\sigma_{0}=median\left\{\left|v_{k}\right|\right\}/0.6475$$

where $S_{k}$ is the standardized residual corresponding to the *k*th measurement or standardized correction for the *k*th predicted state element. Here, c is a constant, usually chosen as 1.0–2.0, and $\sigma_{0}$ is the variance scale factor. The constant 1/0.6745 is a correction factor for Fisher consistency in the Gaussian distribution [35].

When gross errors exist in observation, the adaptive UKF is unable to resist the effect of gross errors. In order to solve the problem, an adaptive robust UKF based on the Sage-Husa filter and robust estimation is proposed. Since the system noise variance matrix ${\hat{Q}}_{k}$ and the measurement noise variance matrix ${\hat{R}}_{k}$ cannot be estimated simultaneously, the proposed adaptive robust UKF applies the Sage-Husa filter to adaptively adjust the system noise variance matrix. At the same time, the equivalent gain matrix based on the Huber function is constructed to eliminate or reduce the effects of gross errors. 

**Figure 1:**
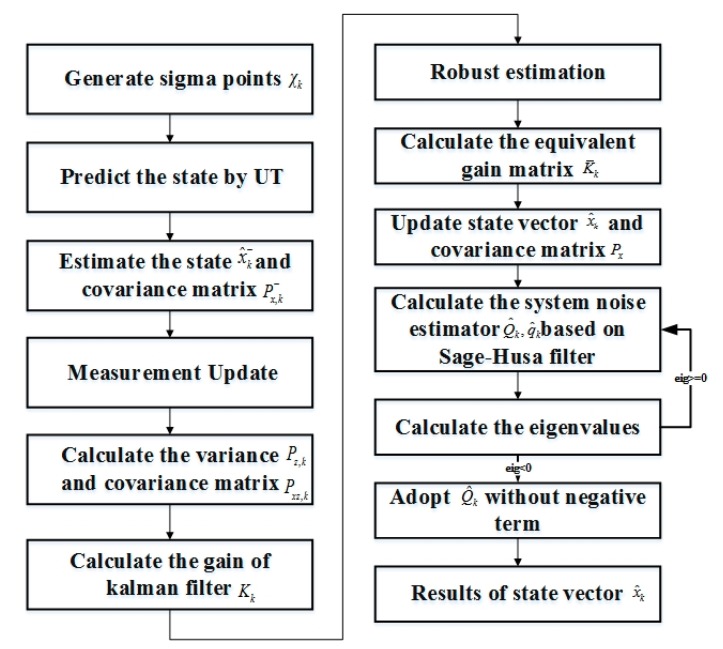
The flowchart of the adaptive robust unscented Kalman filter (UKF).

The flowchart of the proposed adaptive robust UKF is shown in Figure 1. The detailed steps of the algorithm are explained as follows:

(1) The initial position information, speed, turning radius, and turning angle ${\hat{x}}_{0}$ of the AUV are initialized and the sigma points $\chi_{k}$ are generated.

(2) The state is predicted by the UT. Then, the state vector ${\hat{x}}_{k}^{-}$ and the covariance matrix $P_{x,k}^{-}$ are estimated by Equations (9) and (10).

(3) The measurement prediction vector ${\hat{z}}_{k}^{-}$, the variance matrix $P_{z,k}$, and the covariance matrix $P_{xz,k}$ are calculated by Equations (11)–(13).

(4) The gain of the Kalman filter $K_{k}$ is computed by Equation (14). Based on the Kalman filter, the equivalent gain matrix ${\bar{K}}_{K}$ is calculated to achieve robust estimation by Equation (28), while the state vector ${\hat{x}}_{k}$ is calculated by Equation (31).

(31) $${\hat{x}}_{k}={\hat{x}}_{k}^{-}+{\bar{K}}_{K}\left[z_{k}-{\hat{z}}_{k}^{-}\right]$$

(5) The mean of the system noise vector ${\hat{q}}_{k}$ and the system noise variance matrix ${\hat{Q}}_{k}$ are calculated by Equations (17) and (18).

(6) The eigenvalues eig of the system noise variance matrix ${\hat{Q}}_{k}$ is finally calculated. If the  eig≥0, ${\hat{Q}}_{k}$ is calculated by Equation (18); otherwise, ${\hat{Q}}_{k}$ is calculated by Equation (22).

## 4. Experiments and Analysis

To verify the adaptive UKF, the simulated long baseline positioning experiment of the AUV and the real marine experimental data of the ultrashort baseline positioning of an underwater towed body are carried out. Then, the adaptive robust UKF is analyzed and compared in the different situations through the simulation experiment.

### 4.1. Comparison of UKF and Adaptive UKF

#### 4.1.1. Simulation Experiment and Analysis

**Figure 2:**
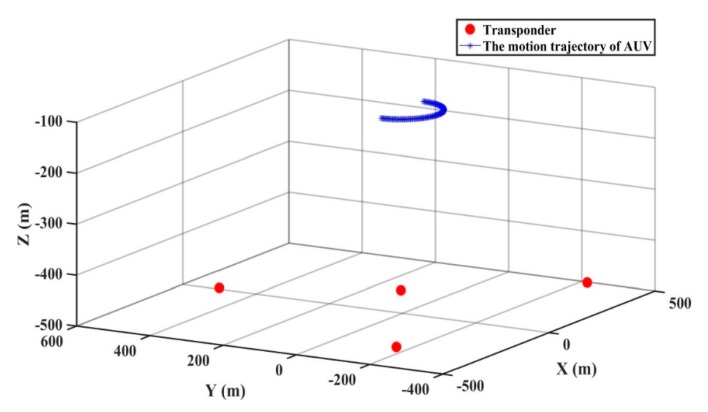
The autonomous underwater vehicle (AUV) motion trajectory and the distribution of the seabed transponders.

The simulation experiment is conducted for AUV positioning. The AUV motion trajectory and the distribution of the seabed transponders are shown in Figure 2. The AUV receives the acoustic signal from the simulated seabed transponders in red, which are centered radially on the seafloor. The coordinates of the four transponders are (0 m, 500 m, −497 m), (435 m, −253 m, −486 m), (−428 m, −232 m, −474 m), and (5 m, 7 m, −456 m), respectively. The blue line represents the uniform turning motion of the AUV at a depth of 100 m, and the motion parameters include the *x* coordinate, *y* coordinate, *z* coordinate, speed, turning radius, and turning angle. The initial position of the AUV is (100 m, 0 m, −100 m), the turning radius is chosen as 100 m, and the turning angle is π/100 rad. A total of 100 epochs are simulated with a sampling interval of 1 s, and the Munk velocity [36] profile and ray tracing algorithm are used to generate the observations. Because the depth of the simulated AUV is the same, the relative depths of the AUV and underwater transponder are used as the known value for calculation. Therefore, the inside of the state vector $x_{k}$ is the *x* coordinate, *y* coordinate, turning angle, speed, and turning radius, respectively The original observations include acoustic travel time between the transducer equipped with the AUV and the seabed transponders, as well as the sound speed profile. Nowadays, the positioning requirement of the AUV in the sea is at the meter level. The high-precision positioning accuracy of AUV using a multi-sensor can reach the decimeter level, completely meeting the requirements of the fields of submarine detection and ocean environmental monitoring. According to the requirements of positioning accuracy, the systematic error and random error are added to the simulated observations, and the slant range error is 0.1 m. The simulated system noise variance matrix is $Q=Q_{0}\times I_{5\times 5}$, with $Q_{0}=0.01\;$ m^2^. Here, $I_{5\times 5}$ is a unit matrix of five rows and five columns. The systematic error formula proposed by Xu et al. is used and can be expressed as [37]:

(32) $$\delta p_{v}=c_{1}+c_{2}\sin\left[\frac{2\left(t-t_{0}\right)}{T_{s}}\pi \right]\;+c_{3}\sin\left[\frac{\left(t-t_{0}\right)}{T_{L}}\pi \right]\;c_{4}[1-\exp\{-\frac{1}{2}\left|\left|x-x^{\prime }\right|\right|/{\left(2\;km\right)}^{2}]$$

where the constant term is $c_{1}=0.1$ m, the short-period internal wave error term is $c_{2}=0.12$ m, the long-period error term is $c_{3}=0.3$ m, the term related to the measurement range is $c_{4}=0.02$ m, the short period of the internal wave is $T_{s}=15\times 60$ s (15 min) and the long period of internal wave is $T_{L}=12\times 3600$ s (12 h), $\left|\left|x-x^{\prime }\right|\right|$ and is the distance between the transducer and the seabed transponder.

The horizontal position difference between the true value and the calculation value, and the corresponding root mean square (RMS) $RMS=\sqrt{\frac{1}{N}\overset{N}{\underset{k=1}{\sum }}{\left(x_{i}-{\hat{x}}_{k}\right)}^{2}}$ are used to evaluate the performance of the UKF and the adaptive UKF algorithm.

**Figure 3:**
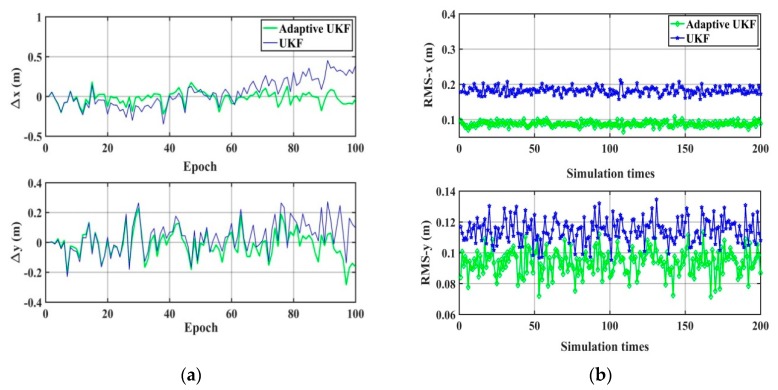
The positioning results of the UKF and the adaptive UKF (Q_0_ = 1 m^2^). (**a**) The difference between the true value and the calculation value; (**b**) The statistical RMS of the *x* direction (RMS-*x*) and the RMS of the *y* direction (RMS-*y*) of 200 simulations.

**Figure 4:**
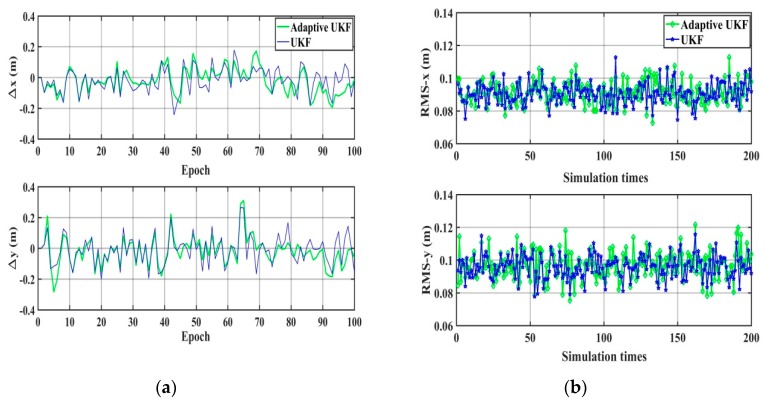
The positioning results of the UKF and the adaptive UKF ($Q_{0}=0.1$ m^2^). (**a**) The difference between the true value and the calculation value; (**b**) The statistical RMS-*x* and RMS-*y* of 200 simulations.

The performance of the UKF and the adaptive UKF algorithm are compared under the condition that there is no gross error except the random error and the systematic error, and the wrong system noise matrix is simulated as $Q=Q_{0}\times I_{5\times 5}$, $Q_{0}=1$ m^2^ or $Q_{0}=0.1$ m^2^. The results are shown in Figure 3 and Figure 4 and Table 1. It can be seen that when the system noise deviates significantly from the true value, for example, amplifying 100 times, the adaptive UKF can adaptively adjust and estimate the system noise, providing higher accuracy positioning results than those of UKF. When the system noise is close to the true value, there is no obvious difference in positioning accuracy between the adaptive UKF and the UKF algorithm.

#### 4.1.2. Real Experiment and Analysis

**Figure 5:**
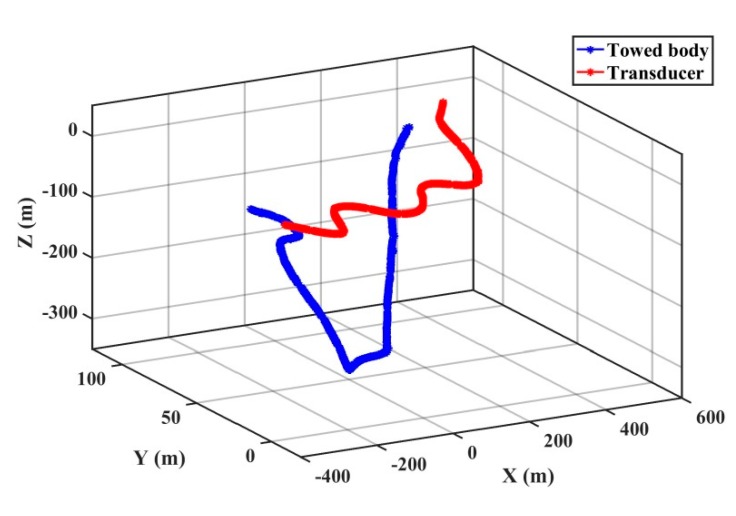
The trajectory of the target motion.

In order to further verify the adaptive UKF, the ultrashort baseline data of the towed body is tested and analyzed for underwater positioning. The towed body refers to the marine integrated survey instrument, including the multibeam sounder, the sub-bottom profiler, and others. The towed body is regarded as the AUV for the real data experiment. The experiment was conducted in the South China Sea in April 2017. The experimental ship is equipped with a global acoustic positioning system (GAPS) ultrashort baseline unit, a differential global positioning system (DGPS), a gyrocompass, a sound velocity profiler (SVP), and an INS. The towed body equipped with the transponder is mounted behind the experimental ship. The position information of the ship can be obtained by the shipboard DGPS and the position of the ship-bottom transducer array can be obtained through coordinate transformation [38]. Data such as the attitude, heading, and speed of the ship are obtained through the gyrocompass and INS. The position of the underwater towed body can be gained in real time through the GAPS ultrashort baseline acoustic positioning system. The mean sound speed measured by the SVP is 1530 m/s. The sampling interval of the INS is 0.01 s and the sampling interval of the GAPS is 1 s. The complete motion trajectory of the experiment is shown in Figure 5, with the red line indicating the position of the transducer and the blue one indicating the position of the transponder. The positioning accuracy is evaluated by the position difference of the abovementioned algorithms and the output value of the instrument, which can be used as references.

**Figure 6:**
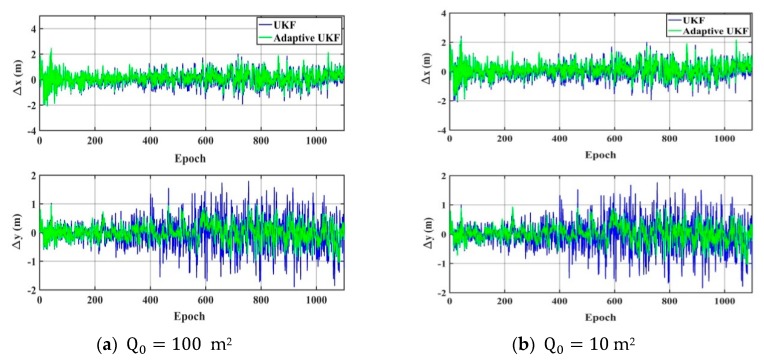
The positioning results of the adaptive UKF algorithm: (**a**) The difference between the calculation value and the reference value in the *x* direction and the *y* direction with the system noise of 100 m^2^; (**b**) The difference between the calculation value and the reference value in the *x* direction and the *y* direction with the system noise of 10 m^2.^

**Figure 7:**
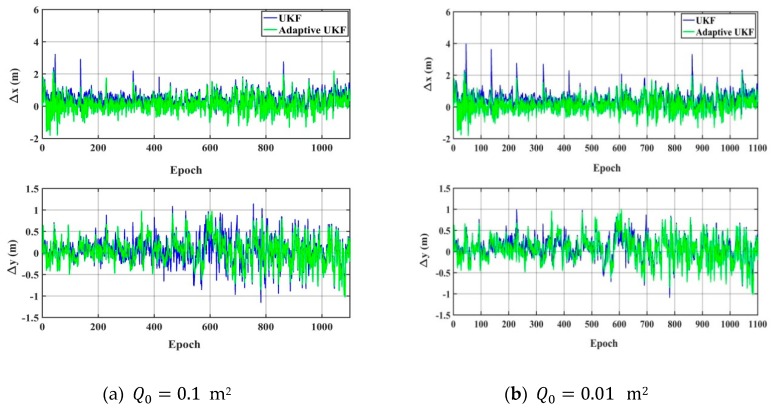
The positioning results of the adaptive UKF algorithm. (**a**) The difference between the calculation value and the reference value in the *x* direction and the *y* direction with the system noise of 0.1 m^2^; (**b**) The difference between the calculation value and the reference value in the x direction and the *y* direction with the system noise of 0.01 m^2^.

**Figure 8:**
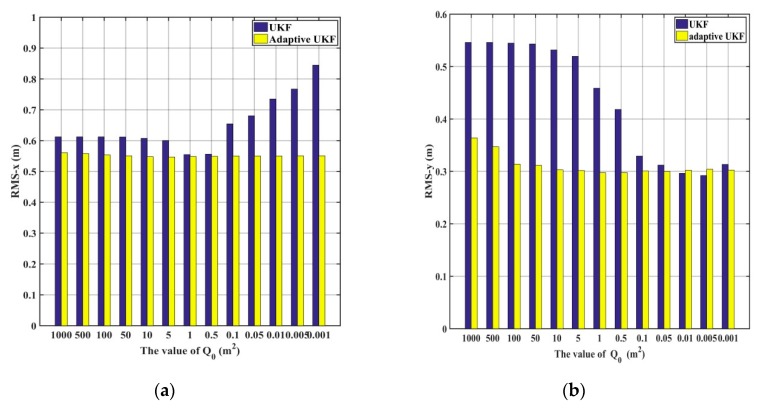
The positioning results of different initial system noises. (**a**) The statistical RMS of the UKF and the adaptive UKF in the *x* direction; (**b**) The statistical RMS of the UKF and the adaptive UKF in the *y* direction.

As shown in Figure 6 and Figure 7, when the system noise is set as $Q_{0}=100\;$m^2^ or $Q_{0}=10\;$m^2^, the accuracy of the UKF obviously decreases in the horizontal direction, the adaptive UKF can adjust the system noise online, and then improve the positioning results. When the system noise is set as $Q_{0}=0.01$ m^2^ or $Q_{0}=0.1$ m^2^, the positioning accuracy of the adaptive UKF is comparable to that of the UKF in the *y* direction, and obviously higher than that of the UKF in the *x* direction. Since the exact system noise is hard to know, we conduct the experiments of the adaptive UKF and the UKF under different system noise levels from 0.001 m^2^ to 1000 m^2^, which are shown in Figure 8 and Table 2. From Figure 8 and Table 2, it can be seen that the accuracy of the UKF changes significantly with different system noise values, while the adaptive UKF can obtain more stable and accurate results with minor influence due to changes in the system noise.

### 4.2. Validation of Adaptive Robust UKF

In this section, the adaptive robust UKF is validated by the simulation experiment of AUV positioning, because the measured data from the ultrashort baseline positioning has only one observation at each observation time, and the validation of the robust algorithm cannot be completed without redundant observations. The experimental trajectory and error simulation of the simulation experiment are the same as in Section 4.1.1. The only difference is that gross errors are simulated based on normal distribution with zero mean and standard deviations of 0.005 s, and added in the acoustic observations every 20 s. At the same time, RMS is used to evaluate the performance of the UKF, the robust UKF, and the adaptive robust UKF.

**Figure 9:**
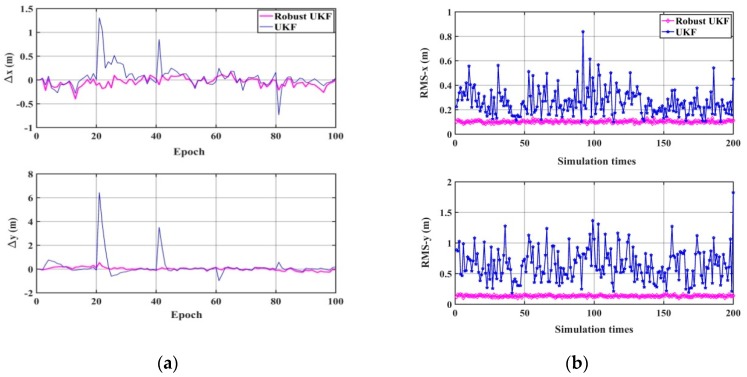
The positioning results of the UKF and the robust UKF. (**a**) The difference between the true value and the calculation value. (**b**) The statistical RMS-*x* and RMS-*y* of 200 simulations.

First, the true system noise is added in the state equation and the measurement errors, including random error, systematic error, and gross error, are added in the acoustic observations. The performances of the UKF and the robust UKF are compared, which are shown in Figure 9 and Table 3. It can be seen that when there are gross errors in the acoustic observations, the UKF can be affected by the gross errors in the observations to obtain unreliable calculation results. The robust UKF can detect gross errors by predicting residuals and can achieve robust estimation by the equivalent gain matrix. Table 3 shows that the mean RMS values of the *x* direction and *y* direction robust UKF are about 0.103 m and 0.135 m, respectively, which are much smaller than those of the UKF (0.264 m and 0.651 m, respectively). The same conclusions can be drawn for the maximum and minimum RMS values for the robust UKF and the UKF. Therefore, the robust UKF can efficiently control the influence of gross errors and considerably improve the positioning accuracy.

**Figure 10:**
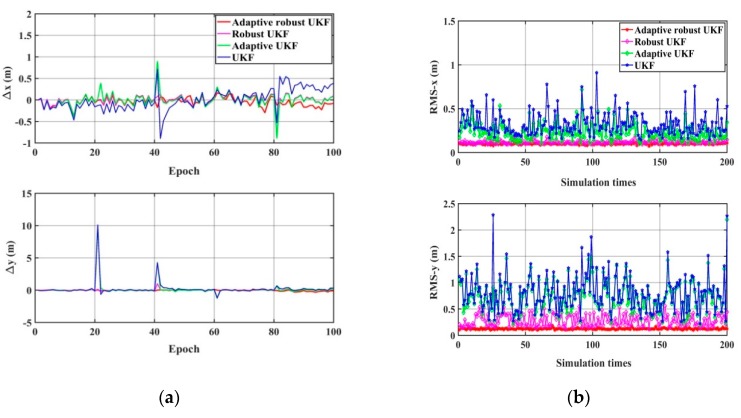
The positioning results of different algorithms ($Q_{0}=1$ m^2^). (**a**) The difference between the true value and the calculation value. (**b**) The statistical RMS-*x* and RMS-*y* of 200 simulations.

**Figure 11:**
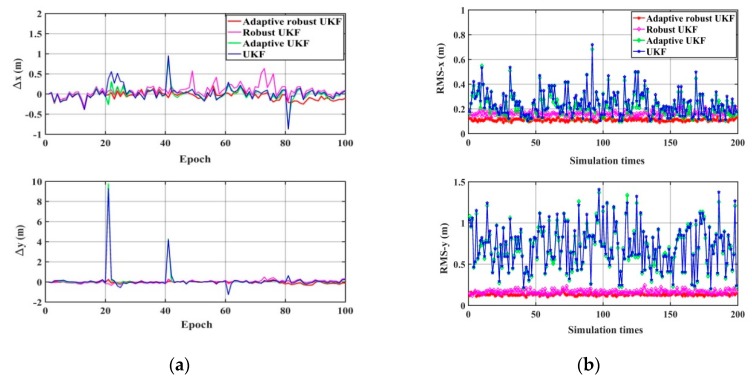
The positioning results of different algorithms ($Q_{0}=0.1$ m^2^). (**a**) The difference between the true value and the calculation value. (**b**) The statistical RMS-*x* and RMS-*y* of 200 simulations.

Then, the performance of the UKF, the adaptive UKF, the robust UKF, and the robust adaptive UKF algorithm are compared under the condition that measurement errors exist, including random error, systematic error, and gross error; and the wrong system noise matrix is simulated as $\;Q=Q_{0}\times I_{5\times 5}$, $Q_{0}=1$ m^2^ or $Q_{0}=0.1$ m^2^. The results are shown in Figure 10 and Figure 11 and Table 4. It can be seen that: (1) the UKF has no ability to resist the influence of gross error and imprecise system noise, both of which reduce its positioning accuracy greatly; (2) the robust UKF can effectively control the influence of gross error by using robust estimation, but the system noise must be known precisely; (3) the adaptive UKF can adaptively adjust and estimate the system noise based on the Sage-Husa filter, but it cannot resist the influence of the gross error; (4) the adaptive robust UKF can estimate system noise using the Sage-Husa filter and achieve robust estimation with the equivalent gain matrix, which is why it can deal with both the problems of unknown noise statistics and gross error, and performs as the best algorithm in terms of positioning accuracy and reliability.

## 5. Conclusions

Because of the complex marine environment, the it is difficult to accurately obtain the system noise, and the acoustic observations usually have gross errors, which influence the positioning accuracy of the classical UKF. Based on the Sage-Husa filter, an adaptive robust UKF algorithm is proposed in this paper. The following conclusions can be drawn from our analysis, computation, and comparison.

(1) He unknown or imprecise system noise can cause the divergence of the classical UKF, so this paper proposes an adaptive UKF based on the Sage-Husa filter. No matter how much the initial system noise is given, the adaptive UKF can accurately estimate the system noise through the time-varying noise estimator. At the same time, the adaptive UKF can avoid the problem of a negative definite value of the system noise variance matrix based on the eigenvalues judgement. 

(2) When there are gross errors in the observation, the adaptive UKF has no ability to resist the effect of gross errors. To solve the problem, this study presents an adaptive robust UKF based on the Sage-Husa filter and the equivalent gain matrix. The adaptive robust UKF can adaptively adjust the system noise to solve the divergence problem and control the effects of gross measurement errors on dynamic state estimates. Compared with the classical UKF and adaptive UKF, the accuracy of the adaptive robust UKF is significantly improved. Therefore, the proposed adaptive robust UKF can provide an effective method for AUV acoustic navigation and positioning.

In actual marine navigation and positioning, if abnormal system noise and measurement noise occur simultaneously, the proposed adaptive robust UKF may provide unsatisfactory results or even lead to filter divergence in this rare situation. Therefore, future research should focus on how to adjust the system noise and the measurement noise at the same time.

## Figures and Tables

**Table 1:** The statistical results of the UKF and the adaptive UKF.

$\mathrm{System}\;\mathrm{Noise}\;Q_{0}\;(m^{2})$	Statistics	Method	RMS-*x* (m)	RMS-*y* (m)
1	Mean	UKF	0.182	0.114
		adaptive UKF	0.088	0.094
	Max.	UKF	0.212	0.134
		adaptive UKF	0.107	0.113
	Min.	UKF	0.158	0.095
		adaptive UKF	0.006	0.071
0.1	Mean	UKF	0.090	0.095
		adaptive UKF	0.091	0.097
	Max.	UKF	0.112	0.115
		adaptive UKF	0.112	0.121
	Min.	UKF	0.074	0.077
		adaptive UKF	0.072	0.075

**Table 2:** The positioning results of different system noise levels.

$\mathrm{The}\;\mathrm{Value}\;\mathrm{of}\;Q_{0}\;(m^{2})$	Method	RMS-*x* (m)	RMS-*y* (m)
1000	UKF	0.612	0.546
	Adaptive UKF	0.561	0.363
500	UKF	0.612	0.546
	Adaptive UKF	0.557	0.347
100	UKF	0.612	0.544
	Adaptive UKF	0.553	0.313
50	UKF	0.611	0.543
	Adaptive UKF	0.550	0.303
10	UKF	0.607	0.531
	Adaptive UKF	0.547	0.303
5	UKF	0.600	0.519
	Adaptive UKF	0.546	0.301
1	UKF	0.554	0.458
	Adaptive UKF	0.548	0.297
0.5	UKF	0.556	0.418
	Adaptive UKF	0.548	0.297
0.1	UKF	0.654	0.329
	Adaptive UKF	0.580	0.300
0.05	UKF	0.680	0.312
	Adaptive UKF	0.550	0.302
0.01	UKF	0.735	0.296
	Adaptive UKF	0.550	0.302
0.005	UKF	0.767	0.292
	Adaptive UKF	0.551	0.304
0.001	UKF	0.844	0.313
	Adaptive UKF	0.551	0.303

**Table 3:** The statistical RMS of the UKF and the robust UKF with 200 simulations.

Statistics	Method	RMS-*x* (m)	RMS-*y* (m)
Mean	UKF	0.264	0.651
	Robust UKF	0.103	0.135
Max.	UKF	0.839	1.827
	Robust UKF	0.130	0.175
Min.	UKF	0.101	0.183
	Robust UKF	0.085	0.102

**Table 4:** The statistical results of different algorithms.

System Noise *Q*_0_ (m^2^)	Statistics	Methods	RMS-*x* (m)	RMS-*y* (m)
1	Mean	UKF	0.324	0.801
		Adaptive UKF	0.232	0.730
		Robust UKF	0.122	0.286
		Adaptive robust UKF	0.101	0.106
	Max.	UKF	0.912	2.287
		Adaptive UKF	0.719	2.197
		Robust UKF	0.196	0.557
		Adaptive robust UKF	0.124	0.289
	Min.	UKF	0.144	0.226
		Adaptive UKF	0.099	0.197
		Robust UKF	0.094	0.121
		Adaptive robust UKF	0.074	0.100
0.1	Mean	UKF	0.248	0.716
		Adaptive UKF	0.226	0.704
		Robust UKF	0.163	0.167
		Adaptive robust UKF	0.112	0.134
	Max.	UKF	0.720	1.408
		Adaptive UKF	0.683	1.370
		Robust UKF	0.224	0.245
		Adaptive robust UKF	0.137	0.196
	Min.	UKF	0.104	0.203
		Adaptive UKF	0.100	0.201
		Robust UKF	0.124	0.121
		Adaptive robust UKF	0.091	0.100
